# Health-related quality of life following lung transplantation for cystic fibrosis: A systematic review

**DOI:** 10.1016/j.clinsp.2023.100182

**Published:** 2023-04-01

**Authors:** Aarohanan Raguragavan, Dujinthan Jayabalan, Akshat Saxena

**Affiliations:** a University of Western Australia School of Medicine, Perth, Australia; b Department of Cardiothoracic Surgery, Fiona Stanley Hospital, Perth, Australia

**Keywords:** Cystic fibrosis, Lung transplantation, Health-related quality of life, Adult lung recipients

## Abstract

**Background:**

Lung transplantation represents the definite treatment for CF patients with advanced-stage pulmonary disease. Recent major developments in the treatment of CF indicate the need for an evaluation of lung transplantation as the current best practice in end-stage disease. This systematic review was performed to evaluate the impact of lung transplantation on health-related quality of life in patients with CF.

**Methods:**

PubMed was searched for studies matching the eligibility criteria between January 2000 and January 2022. OVID (MEDLINE), Google Scholar, and EBSCOhost (EMBASE) as well as bibliographies of included studies were also reviewed. Applying predetermined eligibility criteria, the included studies were selected. Predetermined forms were used to conduct a quality appraisal and implement data tabulation. Results were synthesized by narrative review. This systematic review was prospectively registered in the PROSPERO register (CRD 42022341942).

**Results:**

Ten studies (1494 patients) were included. Lung transplantation results in improvements in HRQoL in CF patients relative to their baseline waitlisted state. Up to five years postoperatively CF patients retain their HRQoL at levels similar to the general population. There are several modulating factors that impact HRQoL outcomes in CF patients post-LTx. Compared to lung recipients with other diagnoses CF patients achieve either greater or equal levels of HRQoL.

**Conclusion:**

Lung transplantation conveys improved HRQoL to CF patients with the advanced-stage pulmonary disease for up to five years, and to levels comparable to the general population and non-waitlisted CF patients. This systematic review quantifies, using current evidence, the improvements in HRQoL gained by CF patients following lung transplantation.

## Highlights

• 10 studies (1494 patients) were included in this systematic review.
• LTx confers improved HRQoL in CF patients relative to their baseline state.
• Up to 5-years post-LTx, CF patients’ HRQoL remains at the general population level.
• Several factors modulate HRQoL outcomes in CF post-LTx.
• HRQoL post-LTx in CF patients is either equal to or greater than other indications.

## Introduction

### Rationale

Cystic fibrosis^1^ is a severely life-shortening disease and the most common autosomal recessive disease in the Caucasian population.^2^^,^^3^ Single-gene mutations in the Cystic Fibrosis Transmembrane Conductance Regulator (CFTR) gene result in airway obstruction and impaired mucociliary clearance and as such characterize the pulmonary component of the disease.^4^ Individuals with CF develop progressive airway inflammation and recurrent respiratory infections, leading to bronchiectasis and chronic respiratory failure.^4^ The pulmonary component of CF is the leading cause of mortality and morbidity in this patient population.^5^

Cystic fibrosis has historically been the third most common indication for lung transplantation^6^^,^^7^ and is considered a crucial management option for patients with advanced cystic fibrosis lung disease.^8^ Patients optimally selected for lung transplantation are shown to experience a net survival benefit.^3^^,^^4^^,^^9^ In recent years the authors have seen significant advancements in the medical management of CF with the advent of the CFTR modulator drug class,^10^^,^^11^ resulting in improvements in Health-Related Quality of Life (HRQoL).^12^ Improvements in long-term outcomes are yet to be published for these new medications and may alter the timing for referral or listing for transplantation.

Given advances in management it is imperative that up-to-date evidence regarding post-transplant outcomes such as HRQoL be quantified and reported so as to best guide clinician judgment in the management of patients with CF. A systematic review of the literature is fundamental to enabling informed clinical judgment and directing future research. This systematic review aims to: (i) Summarise the literature and clarify strengths and weaknesses of current evidence on HRQoL outcomes post-LTx in CF patients, (ii) Demonstrate and quantify the changes in HRQoL over time following LTx in CF patients with advanced cystic fibrosis lung disease, and (iii) Provide a foundation for future research into HRQoL in CF patients.

## Methods

The PRISMA Guidelines were used to structure this systematic review.^13^, ^14^, ^15^ This systematic review was prospectively registered in the PROSPERO register (CRD 42022341942).

### Definition and measurement of HRQoL

The importance of HRQoL as a measure is evident when understanding that advancements in medicine often lead to the extension of life at the cost of quality of life or improve quality of life without extending life.^16^ HRQoL can be simply defined as the measure of an individual's perception of how well they function in life.^17^ Hays et al. further expand their definition to include that HRQoL consists of objective measures of social, mental, and physical functioning in addition to internal subjective perceptions of quality of life.^17^

Studies assessing HRQoL following lung transplantation for cystic fibrosis employ a wide variety of both disease-specific and generic HRQoL measurement instruments. Hays et al. identify the characteristics of a good HRQoL measurement tool to be the inclusion of a conceptual model, reliability, validity, and the reporting of minimally important differences and interpretations of scores.^17^ The Cystic Fibrosis Quality of Life Questionnaire (CFQoL)^18^ represents a widely used pulmonary disease-specific instrument, which differs from the St. George Respiratory Questionnaire (SGRQ)^19^^,^^20^ a pulmonary-specific HRQoL instrument. Examples of generic HRQoL instruments include the Medical Outcomes Survey Short-Form-36 (SF-36)^20^^,^^21^ and the EuroQol-5D (EQ5D).^20^^,^^22^

In order to accurately evaluate lung transplantation as a management option for patients with cystic fibrosis adequate HRQoL instruments, which cover all domains, and are consistently reliable and valid, must be used.^17^

### Eligibility criteria

The following characteristics were necessary to be eligible for inclusion in this review: (i) Adult patients with CF receiving primary lung transplantation, (ii) Recording of disease-specific, pulmonary-specific, and/or generic HRQoL data using a validated instrument post-LTx, and (iii) Comparison of postoperative HRQoL scores to pretransplant waitlisted/non-waitlisted patients, the general population, and/or other disease indications for lung transplantation HRQoL scores. For inclusion in this systematic review studies were required to have the following characteristics: (i) Publication date after January 2000, (ii) English language, and (iii) Original articles. Only original search manuscripts published in English in peer-reviewed journals were included.

### Information sources and search strategy

**Fig. 1:**
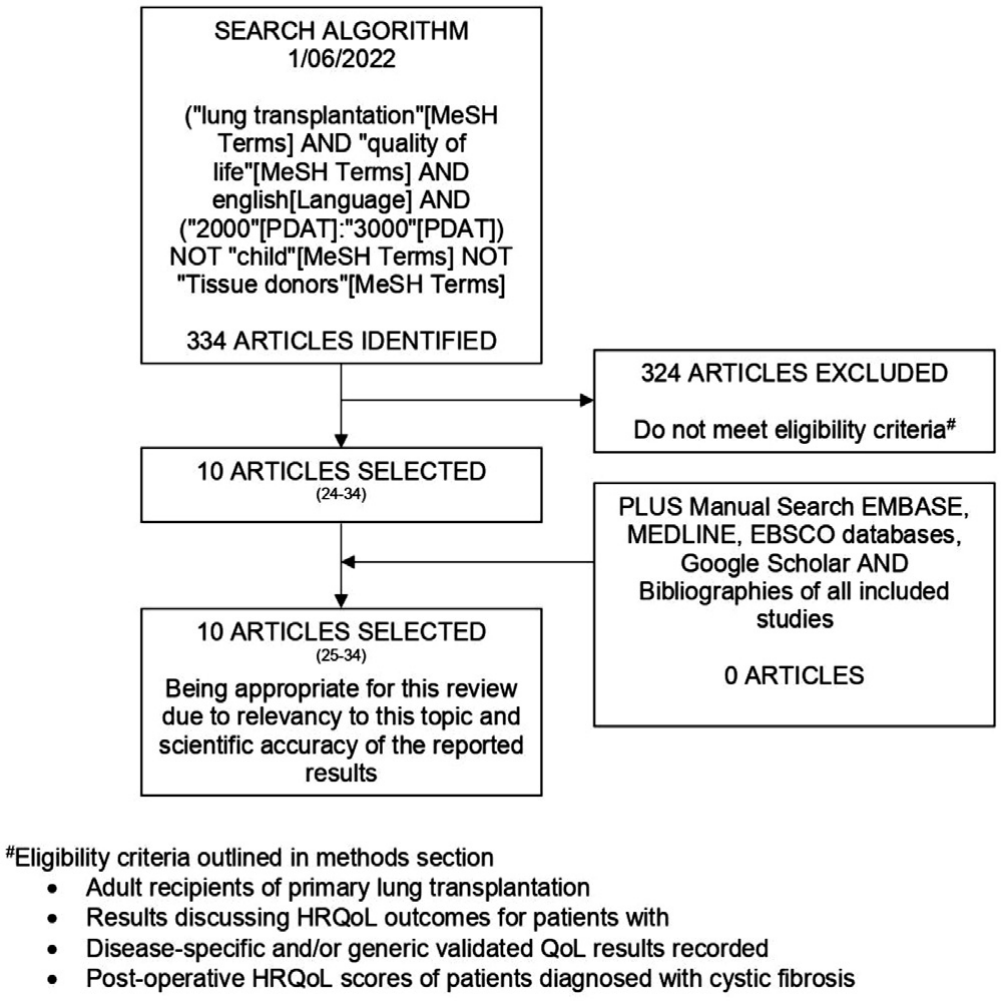
Search algorithm.

In February 2022, A.R. performed a literature search using a MeSH keyword search on PubMed (MEDLINE) for studies that matched the above eligibility criteria (Fig. 1). Additionally, OVID (MEDLINE), Google Scholar, SciELO, and EBSCOhost (EMBASE), as well as bibliographies of each included study, were manually searched to recover studies not included in the initial MeSH keyword search. All identified articles were retrieved from said databases.

### Study selection

A.R. independently screened titles and abstracts of studies retrieved from the MeSH keyword and manual searches. Studies were not included if they did not meet eligibility criteria. Consensus for studies included for review was achieved by discussion between A.R., D.J., and A.S. based on the predetermined eligibility criteria.

### Data items and extraction

Data items for assessment of study quality (Table 1) and study results (Table 2) were predetermined. Data extraction was then performed by D.J. using standardized pilot forms.

**Table 1:** Quality appraisal.

Year	Author	N° patients	Study design	Generic HRQoL instrument	Lung specific measures	Participation rate response rate	Level of evidence	Patient demographics				
								Age (mean ± SD or range)	Sex	Location	Aetiology	Other
2004	Gee et al.^33^	223	R	‒	CFQoL	PR: NR	IV	25.1±7.1	45.7% M	United Kingdom	CF 223	13 on waitlist, 14 post-transplant
						RR: 57%						
2004	Vermeulen et al.^27^	215	P	NHP	‒	PR: NR	III	Mean (range)	CF: 53% M	The Netherlands	COPD 39, α1-antitrypsin deficiency 51, Bronchiectasis 17, Pulmonary fibrosis 20, Pulmonary hypertension 35, Miscellaneous 21	SLTx excluded
						RR: 20.9%		CF: 29 (18‒55)	Non-CF: 44% M			
								Non-CF: 45 (19‒60)				
2005	Smeritschnig et al.^28^	94	R	SF-36	SGRQ	PR: NR	IV	Mean±SD (range)	48% M	Austria	COPD/emphysema 50, Fibrosis 16, Pulmonary vascular disease 13, Cystic Fibrosis 10, Other 5	
						RR: 87%		51±10 (22‒69)				
2005	Vasiliadis et al.^32^	105	R	SF-36	‒	PR: 78%	IV	Mean age at transplant: 41.42±13.23	37% M	Canada	COPD 29, CF 22, Bronchiectasis 7, Restrictive diseases 8, Pulmonary vascular diseases 5	33 SLTx, 38 BLTx
						RR: NR						
2013	Copeland et al.^30^	131	P	SF-36	‒	PR: 96%	III	55 (45‒61) (25^th^‒75^th^)	51% M	USA	COPD 66, CF 22, Idiopathic pulmonary fibrosis 28, Other 15	87 BLTx, 44 SLTx
						RR: NR						
2014	Dębska et al.^34^	45	R	‒	CFQoL	PR: 100%	IV	NR	NR	Poland	CF: 45	10 post-transplant, 15 on waitlist (unstable), 20 not on waitlist not transplanted (stable)
						RR: NR						
2015	Singer et al.^31^	387	P	SF-36	SGRQ	PR: 45.8%	III	Median (range)	All transplanted 55% M	Canada	All transplant	50 SLTx, 325 BLTx, 12 HLTx
						RR: 84.2%		All: 54 (18‒74)			Cystic fibrosis 83, Interstitial lung disease 129, COPD 108, Pulmonary hypertension 35, other 32	
								CF: 83 (22)				
									All Post trans data 55% M			Post-transplant: 42 SLTx, 274 BLTx, 10 HLTx
											Post trans data	
											Cystic fibrosis 71, Interstitial lung disease 106, COPD 96, Pulmonary hypertension 27, other 26	
2016	Singer et al.^29^	211	P	SF12-PCS and MCS, and EQ5D	AQ20-R	PR: 85%	III	Median (IQR)	Overall: 54% M	USA	COPD 36, Pulmonary arterial hypertension 8, Cystic fibrosis 19, Pulmonary fibrosis 148	
						RR: 32%		Overall: 58 (48, 64)	CF: 42% M			
								CF: 28 (24, 40)				
2020	Perez et al.^25^	23	P	SF12-PCS and MCS, and EQ5D	AQ20-R	PR: 76.7%	III	Mean±SD	CF: 49% M	USA	CF: 30	
						RR: NR		CF: 31.0±7.9			Not CF: 362	
2020	Stacel et al.^26^	60	R	WHOQOL-BREF	SGRQ	PR: 34%	IV	Overall mean: 39.5	51.7% M	Poland	Cystic fibrosis 15, COPD 15, Idiopathic pulmonary arterial hypertension 6, Interstitial lung disease 20: (idiopathic pulmonary fibrosis 12, sarcoidosis 2, histiocytosis 3, lymphangioleiomyomatosis 1, hypersensitivity pneumonitis 2), Other 4 (Osler-Weber-Rendu syndrome 1, Williams-Campbell syndrome 1, Pulmonary veno-occlusive disease 1, bronchiectasis 1)	Overall: 20 SLTx, 40 BLTx
						RR: NR		SLTx: 45.65±12.97				CF: 2 SLTx,13 BLTx
								BLTx: 36.45±14.77				

AQ20, Airways questionnaire 20; BLTx, Bilateral Lung Transplant; CFQoL, The Cystic fibrosis Quality of Life; COPD, Chronic Obstructive Pulmonary Disease; CF, Cystic Fibrosis; EQ5D, EuroQol 5D; HLTx, Heart-Lung Transplant; ILD, Interstitial Lung Disease; IPF, Idiopathic Pulmonary Fibrosis; M, Male; MCS, Mental Component Summary Score; NHP, Nottingham Health Profile; NR, Not Reported; PCS, Physical Component Summary Score; PR, Participation Rate; RR, Response Rate; SF12, 12-item Short Form survey; SF-36, 36-item Short Form survey; SD, Standard Deviation, SGRQ, St. George Respiratory Questionnaire; SLTx, Single Lung Transplant; USA, United States of America, WHOQOL-BREF, World Health Organisation quality of life Brief Questionnaire.

**Table 2:** Results of included studies.

Year	Author	Method of follow-up	Follow-up interval	Comparison groups		
				Pre-transplant	Other groups	Other time periods
2004	Gee^33^	Self-administered questionnaire by mail	NR	‒	CF non-transplanted patients	‒
		The median values (25th percentile–75th percentile) if CFQoL domains of patients not on the waiting list (*n* = 196), on the waiting list (*n* = 13) and post-transplant (*n* = 14)				
		Not on waiting list: physical functioning 91 (76–98), social functioning 95 (80–100), treatment issues 80 (60–93), chest symptoms 70 (50–90), emotional functioning 85 (67–92), concerns for the future 43 (26–62), interpersonal relationships 66 (46–80), body image 66 (46–86), career issues 60 (40–85)				
		Waiting list: physical functioning 66 (55–75), social functioning 70 (52–90), treatment issues 53 (40–70), chest symptoms 50 (30–70), emotional functioning 75 (48–87), concerns for the future 33 (21–61), interpersonal relationships 52 (39–74), body image 60 (36–73), career issues 35 (27–62)				
		Post–transplant: physical functioning 96 (92–100), social functioning 95 (93–100), treatment issues 96 (91–100), chest symptoms 100 (77–100), emotional functioning 91 (82–98), concerns for the future 51 (25–66), interpersonal relationships 62 (39–74), body image 66 (45–75), career issues 37 (33–75)				
		Multiple linear regression analysis using beta estimates (95% CI) was utilised to demonstrate that post–transplant status was correlated with HRQoL. In the CFQoL domains most related to functional health status: physical functioning 8.2 (-0.93, 14), social functioning 7.3 (-0.36, 13), treatment issues 16 (6.0, 23) and chest symptoms 20 (10.28), patients reported a much higher HRQoL post lung transplantation.				
2004	Vermeulen^27^	Self-administered questionnaires sent by mail	Every 3-months while on the waitlist. On admission to waitlist, at 1-months, 4-months, 7-months and 6-monthly thereafter until 31-months post-Lung transplantation	‒	Patients with other diagnoses (non-CF)	Compared to months 4, 7, 19, 31, 43, and 55 post-transplant
		When compared to the general population reference value, before undergoing a lung transplantation, CF patients had significantly worse HRQoL in the NHP domains: mobility (p < 0.001) and energy (p = 0.001). On the other hand, the non-CF patients had significantly worse HRQoL in the NHP domains (p < 0.03): energy, sleep, social (isolation) and emotional (reaction). Between 1- to 4-months after transplantation, when compared to non-CF patients, CF patients experienced significantly greater HRQoL in the NHP domains: mobility (p < 0.001) and energy (p = 0.003). There was no significant difference between NHP domains (p > 0.05): pain, sleep, social (isolation) and emotional (reaction). 4-months after transplantation, when compared to non-CF patients, CF patients experienced significantly greater HRQoL in the NHP domain: mobility (p = 0.03). There was no significant difference between the NHP domains: pain, energy, sleep, social (isolation) and emotional (reaction). At 7-months (p = 0.03) and 13-months (p = 0.01) post transplantation, CF patients experienced significantly greater HRQoL in the NHP domain: sleep, but there was no significant difference between the NHP domains: mobility, pain, energy, social (isolation) and emotional (reaction).				
		NHP scores (median (range) for CF vs. non-CF for time periods: Before Lung transplantation, 1-month, 4-months, 7-months, and 13-months across all domains)				
		Mobility: CF: 33 (0–79) vs. non–CF: 57 (10–89), CF: 22 (0–60) vs. non–CF: 22 (0–88), CF: 0 (0–0) vs. non–CF: 0 (0–57) (p = 0.03), Mobility: CF: 0 (0–0) vs. non–CF: 0 (0–35), Mobility: CF: 0 (0–11) vs. non–CF: 0 (0–57)				
		Pain: CF: 0 (0–57) vs. non–CF: 0 (0–91), Pain: CF: 0 (0–13) vs. non–CF: 10 (0–67), Pain: CF: 0 (0–0) vs. non–CF: 0 (0–72), Pain: CF: 0 (0–0) vs. non–CF: 0 (0–94), Pain: CF: 0 (0–17) vs. non–CF: 0 (0–84)				
		Energy: CF: 61 (0–100) vs. non–CF: 100 (0–100), Energy: CF: 0 (0–61) vs. non–CF: 0 (0–100), Energy: CF: 0 (0–0) vs. non–CF: 0 (0–100), Energy: CF: 0 (0–0) vs. non–CF: 0 (0–63), Energy: CF: 0 (0–39) vs. non–CF: 0 (0–100)				
		Sleep: CF: 13 (0–100) vs. non–CF: 14 (0–100) (p = 0.03), Sleep: CF: 13 (0–35) vs. non–CF: 34 (0–87) (p = 0.03), Sleep: CF: 6 (0–38) vs. non–CF: 13 (0–100), Sleep: CF: 6 (0–13) vs. non–CF: 13 (0–87) (p = 0.03), Sleep: CF: 6 (0–13) vs. non–CF: 13 (0–100) (p = 0.01)				
		Social (isolation): CF: 0 (0–65) vs. non–CF: 0 (0–100), Social (isolation): CF: 0 (0–22) vs. non–CF: 0 (0–84), Social (isolation): CF: 0 (0–0) vs. non–CF: 0 (0–65), Social (isolation): CF: 0 (0–22) vs. non–CF: 0 (0–65), Social (isolation): CF: 0 (0–22) vs. non–CF: 0 (0–41)				
		Emotional (reaction): CF: 9 (0–47) vs. non–CF: 14 (0–100), Emotional (reaction): CF: 0 (0–24) vs. non–CF: 4 (0–67), Emotional (reaction): CF: 0 (0–10) vs. non–CF: 0 (0–74), Emotional (reaction): CF: 0 (0–21) vs. non–CF: 0 (0–70), Emotional (reaction): CF: 0 (0–10) vs. non–CF: 0 (0–33)				
		At 25- and 31-months after transplantation, there was no significant difference between the restrictions on the domains of mobility, pain, energy, sleep, social (isolation) and emotional (reaction). It can be concluded that Lung transplantation has a substantial positive effect on HRQoL in CF and in non-CF patients. HRQoL improved post-transplant, especially the NHP domain of mobility in both groups. CF patients experienced fewer restrictions on several HRQoL domains before transplantation and reached the same level after transplantation compared to non-CF patients may lead to the conclusion that patients with other diagnoses gained more improvement after transplantation compared to CF.				
2005	Smeritschnig^28^	Self-administered questionnaire via a study kit given during their check-up visit to the outpatient department	42 ± 30 months (Range: 3–117 months)	–	Normative healthy and chronic lung disease populations and patients with other diagnoses (non-CF)	–
		SGRQ (compared to individuals with lung disease)				
		Symptoms: 21.1 ± 18.5 vs. Reference value: 62.5 ± 15.5 (p < 0.001)				
		Activity: 36.9 ± 25.0 vs. Reference value: 55.5 ± 24.0 (p < 0.001)				
		Impacts: 20.7 ± 18.3 vs. Reference value: 37.4 ± 22.5 (p < 0.001)				
		Total: 24.4 ± 18.3 vs. Reference value: 47.6 ± 19.7 (p < 0.001)				
		SF-36 domains (compared to healthy reference)				
		Physical function: 68.6 ± 24.1 vs. Reference value: 85.7 ± 22.1 (p < 0.001)				
		Role physical: 59.3 ± 41.9 vs. Reference value: 83.7 ± 31.7 (p < 0.001)				
		Bodily pain: 72.1 ± 28.1 vs. Reference value: 79.1 ± 27.4 (p < 0.05)				
		General health: 58.9 ± 19.8 vs. Reference value: 68.0 ± 20.1 (p < 0.001)				
		Vitality: 59.5 ± 21.9 vs. Reference value: 63.3 ± 18.5d (p = NS)				
		Social function: 82.7 ± 23.1 vs. Reference value: 88.8 ± 18.4 (p < 0.05)				
		Role emotional: 67.8 ± 43.7 vs. Reference value: 90.3 ± 25.6 (p < 0.001)				
		Mental health: 71.4 ± 20.7 vs. Reference value: 73.9 ± 16.4 (p = NS)				
		Physical component summary scale: 43.9 ± 10.1 vs. Reference value: 50.2 ± 10.2 (p < 0.001)				
		Mental component summary scale: 49.8 ± 11.5 vs. Reference value: 51.5 ± 8.1 (p = NS)				
		SF-36 domains (compared to healthy reference)				
		Physical function: 68.6 ± 24.1 vs. Reference value: 85.7 ± 22.1 (p < 0.001)				
		Role physical: 59.3 ± 41.9 vs. Reference value: 83.7 ± 31.7 (p < 0.001)				
		Bodily pain: 72.1 ± 28.1 vs. Reference value: 79.1 ± 27.4 (p < 0.05)				
		General health: 58.9 ± 19.8 vs. Reference value: 68.0 ± 20.1 (p < 0.001)				
		Vitality: 59.5 ± 21.9 vs. Reference value: 63.3 ± 18.5d (p = NS)				
		Social function: 82.7 ± 23.1 vs. Reference value: 88.8 ± 18.4 (p < 0.05)				
		Role emotional: 67.8 ± 43.7 vs. Reference value: 90.3 ± 25.6 (p < 0.001)				
		Mental health: 71.4 ± 20.7 vs. Reference value: 73.9 ± 16.4 (p = NS)				
		Compared to a normative, healthy population, patients who undergo lung transplantation have significantly worse SF-36 domain physical function (p < 0.001) but show better vitality and mental health. SGRQ scores improved significantly (p < 0.001) in all domains (symptoms, activity, impacts and total) when comparing post lung transplantation patients with patients with pre-existing lung disease (COPD/emphysema, Fibrosis, Pulmonary vascular disease, and others). CF patients had the highest SF-36 PCS score amongst all other diagnosis, but the difference was not statistically significant upon analysis of variance. Upon exclusion of the ‘other’ diagnostic category, CF patients had the highest SF-36 MCS score, and a statistically significant difference (p = 0.001) was noted between the different diagnoses during analysis of variance. Viewing these results collectively, CF patients appear to have the best HRQoL post lung transplantation.				
		Diagnosis and quality of life (mean±SD)				
		SGRQ total scores: COPD/emphysema 26.5 ± 19.3, Fibrosis 34.4 ± 17.3, Pulmonary vascular disease 20.4 ± 13.7, CF 7.2 ± 7.4, Others 16.9 ± 10.5 (p = 0.002)				
		SF-36 physical component summary scale: COPD/emphysema 41.7 ± 10.1, Fibrosis 39.6 ± 9.8, Pulmonary vascular disease 48.4 ± 7.4, CF 53.9 ± 6.6, Others 49.0±6.5 (not significant)				
		SF-36 mental component summary scale: COPD/emphysema 48.9 ± 12.3, Fibrosis 48.8 ± 12.3, Pulmonary vascular disease 48.1 ± 10.1, CF 53.4 ± 8.1, Others 58.9 ± 2.9 (p = 0.001)				
2005	Vasiliadis^32^	Self-administered questionnaire by email + follow up telephone call	Grouped into 1, 2, 3, 4 and 5 years		Obstructive airways disease	
		The positivity (increase in scores) of the CF and bronchiectasis group for each domain of SF-36 when compared to the Obstructive Airways Disease (OAD) group was reported. CFB had significantly improved HRQoL scores (p < 0.05) in all domains except for physical function (p > 0.05).				
		Determinants of the domains of the SF-36, mean effect change relative to OAD (95% CI), (p-value)				
		Physical function: (p > 0.05)				
		Role physical: 23 (5, 41), (p < 0.05)				
		Bodily pain: 32 (15, 50), (p < 0.05)				
		General health: 18 (5, 32), (p < 0.05)				
		Vitality: 25 (15, 35), (p < 0.05)				
		Social function: 37 (21, 53), (p < 0.05)				
		Role emotional: 30 (11, 48), (p < 0.05)				
		Mental health: 14 (7, 22) (p < 0.05)				
2013	Copeland^30^	Questionnaire administered at follow up clinic	3, 6, 9 and 12-months		-	-
		The SF-36 PCS score increased by 10.9-points on average from baseline (p < 0.0001) when considering all time points post lung transplantation, comparable to the USA population norms. The first 3-months post lung transplantation had the most significant rise in SF-36 PCS scores (p = 0.03). The SF-36 MCS score did not change on average from baseline (p = 0.36) and was far below the US population norm. The SF-36 MCS score plateaued within the first-year post lung transplantation (p = 0.92). Of all lung disease groups (COPD, IPF, other), CF patients had the highest HRQoL prior to lung transplantation, albeit there was no statistically significant difference (p = 0.43, CF = 37.0, COPD = 34.3, IPF = 34.8, other = 32.3). At 1-year post lung transplantation, the difference in HRQoL from baseline in CF patients, who increased by 14.0 points on average, was significantly the greatest when compared with COPD and IPF groups which increased 10.2 and 7.2 points respectively (p = 0.04).				
2014	Dębska^34^	Questionnaire administered	3 to 37-months post-transplantation	Yes	Patients with CF who are stable (not on the waitlist)	‒
		Of all 3 groups, the post lung transplantation group had mean scores > 50 in all domains of the CFQoL and had the highest mean CFQoL score in the domains: chest symptoms, treatment issues, emotional functioning and in physical functioning, with the social functioning group also being very high. The lowest HRQoL scores in the post lung transplantation group was reported in the CFQoL domains: career concerns, future concerns, body image and interpersonal relationships. The waitlist group had mean scores < 50 in all CFQoL domains, other than emotional function ing and future concerns. When comparing the post lung transplantation group with the waitlist group and the stable CF group, the treatment domain was significantly higher (p < 0.001).				
		CFQoL scores (mean ± SD) across domains for post transplantation, waitlist, and stable CF groups				
		Physical functioning 90.6 ± 8.5, 34.1 ± 22.0, 84.2 ± 15.5				
		Social functioning 73.0 ± 18.4, 34.8 ± 22.5, 81.1 ± 17.0				
		Treatment issues 95.3 ± 8.9, 39.8 ± 19.4, 68.1 ± 20.1				
		Chest symptoms 87.3 ± 12.8, 43.7 ± 19.0, 76.6 ± 15.7				
		Emotional functioning 56.0 ± 11.8, 55.0 ± 17.6, 77.6 ± 19.6				
		Future concerns 60.6 ± 13.2, 55.0 ± 20.6, 51.9 ± 19.2				
		Interpersonal relationships 69.3 ± 17.8, 47.5 ± 14.6, 63.2 ± 23.5				
		Body image 69.3 ± 17.8, 41.7 ± 23.8, 64.6 ± 23.2				
		Career concerns 55.0 ± 10.0, 32.0 ± 15.4, 65.3 ± 25.8				
		Global quality of life 73.9 ± 7.9, 42.6 ± 12.4, 70.8 ± 15.7				
		Using the Kruskal-Wallis test and Dunn post-hoc test, the CFQoL scores (median [IQR], p-value) were compared across domains. Between the 3 groups, a significant difference (p < 0.05) was identified in the CFQoL domains: physical functioning, social functioning, treatment issues, chest symptoms, emotional functioning, body image, career concerns and global quality of life. No significant difference was identified between the CFQoL domains: future concerns and interpersonal relationships. The post lung transplantation group had significantly higher average scores in all domains when compared to the waitlist group (p < 0.05), with the exceptions of the domains: future concerns and interpersonal relationships.				
		Comparison of CFQoL scores (p-value, median [IQR]) across domains for post-transplant, waitlist, and stable CF				
		Physical functioning: p < 0.0011, 91 (82.4, 100), 28 (22, 54), 88 (76, 94)				
		Social functioning: p < 0.0012, 77.5 (60, 85), 32.4 (15, 50), 85(70,95)				
		Treatment issues: p < 0.0013, 100 (93.3, 100), 37.3 (20,60), 66.7 (60, 80)				
		Chest symptoms: p < 0.0014, 100 (100, 100), 45 (30, 55), 80 (65, 85)				
		Emotional functioning: p = 0.0015, 91.3 (75, 100), 52.5 (40, 70), 77.5 (62.5, 97.5)				
		Future concerns: p = 0.469, 55 (46.7, 66.7), 40 (36.7, 66.7), 50 (36.7, 70)				
		Interpersonal relationships: p = 0.057, 65 (52, 70), 44 (36, 58), 66 (44, 78)				
		Body image: p = 0.0086, 66.7 (53.5, 93.3), 40 (20, 53.3), 66.7 (40, 80)				
		Career concerns: p = 0.0017, 55 (50, 65), 30 (20, 50), 70 (40, 85)				
		Global quality of life: p < 0.0018, 74.2 (67.3, 80.8), 38.8 (35.4, 48.8), 73.1 (52.3, 80.4)				
		p-values for comparison of the CFQoL scores median (IQR) between groups across domains for post-transplant vs. waitlist, post-transplant vs. stable CF, waitlist vs. stable CF				
		Physical functioning: p < 0.01, p > 0.999, p < 0.01				
		Social functioning: p = 0.009, p ≥ 0.999, p < 0.001				
		Treatment issues: p < 0.001, p = 0.020, p = 0.012				
		Chest symptoms: p < 0.001, p = 0.066, p = 0.002				
		Emotional functioning: p = 0.001, p = 0.638, p = 0.012				
		Body image: p = 0.023, p > 0.999, p = 0.023				
		Career concerns: p = 0.024, p > 0.999, p < 0.001				
		Global quality of life: p < 0.001, p > 0.99, p < 0.001				
2015	Singer^31^	Questionnaire at routine clinic visits	3 to 12-months post-transplant	‒	Patients with other diagnoses (non-CF)	‒
		Of all diagnostic categories, CF experienced the significantly highest QALY (p < 0.05). When compared to patients with interstitial lung disease, CF patients experienced significantly improved HRQoL (p < 0.05). In the SGRQ score there was an 8-unit difference and in the SF-36 PCS there was a 4-unit difference (95% CI, 2‒7). No significance difference was identified between CF and COPD or pulmonary hypertension in both the SGRQ and SF-36 scores.				
		HRQoL measures by recipient diagnosis without imputing missing values				
		SGRQ (median [IQR]) (p = 0.0006): CF -49.5 (-53.5, -45.5), COPD -49.9 (-53.3, -46.5) ILD -41.1 (-44.4, 37.9) PAH -42.6 (-48.8, -36.3), Other -49.4 (-55.9, -42.8)				
		SF36-PCS (median [IQR]) (p = 0.03): CF 19.6 (17.5, 21.8), COPD 18.3 (16.4, 20.1) ILD 15.4 (13.6, 17.1) PAH 18.0 (14.6, 21.3), Other 17.4 (13.9, 21)				
		SF36-MCS (median [IQR]) (p = 0.2): CF 9.1 (6.7, 11.5), COPD 8.4 (6.4, 10.4) ILD 5.9 (4.0, 7.8) PAH 7.7 (4.0, 11.3), Other 8.1 (4.2, 12)				
		EQ5D (median [IQR]) (p = 0.3): CF 0.28 (0.22, 0.33), COPD 0.30 (0.26, 0.35) ILD 0.25 (0.20, 0.30) PAH 0.22 (0.14, 0.30), Other 0.31 (0.22, 0.40)				
		Over the first 5-years post lung transplantation, using univariate analysis, a significant difference (p = 0.03) was identified between the predicted Quality-Adjusted Life-Year (QALY) across the diagnostic categories, mean (95% CI): CF 2.87 (2.53‒3.20), COPD 2.33 (2.03‒2.63), Interstitial lung disease 2.17 (1.90‒2.44), Pulmonary arterial hypertension 2.53 (2.02‒3.04) and other 2.31 (1.77‒2.85). Using multivariate regression, however, there was no significant correlation between diagnostic categories and QALYs after age adjustment (p = 0.68).				
2016	Singer^29^	Questionnaire at an outpatient study facility	Pretransplant, 3, 6 and 6-monthly thereafter until 36-months.	-	Patients with other diagnoses (non-CF)	-
		No significant difference between CF patients and other diagnostic categories (COPD, pulmonary arterial hypertension, pulmonary fibrosis) were noted before lung transplantation in all HRQoL instruments.				
		SF12-PCS (median [IQR]): COPD 22.0 (18.4, 28.3), Pulmonary arterial hypertension 25.3 (22.8. 31.1), CF 24.1 (14.8, 25.9), Pulmonary fibrosis 22.9 (16.1, 25.7)				
		SF12-MCS (median [IQR]): COPD 52.6 (47.3, 58.5), Pulmonary arterial hypertension 48.8 (28.9, 62.3), CF 44.1 (37.8, 52.6), Pulmonary fibrosis 50.9 (40.6, 57.1)				
		AQ20-R (median [IQR]): COPD 6 (3, 11), Pulmonary arterial hypertension 7 (2, 12), CF 5 (4, 7), Pulmonary fibrosis 7 (4, 9)				
		EQ5D (median [IQR]): COPD 0.69 (0.59, 0.79), Pulmonary arterial hypertension 0.79 (0.60, 0.82), CF 0.60 (0.44, 0.78), Pulmonary fibrosis 0.69 (0.55, 0.78)				
		A significant difference between the HRQoL (from pretransplant to 3-months posttransplant) of different diagnostic categories was identified after adjusting for age and other relevant cofactors (sex, baseline BMI, FEV1, 6-minute walk distance and Lung Allocation Score [LAS]). CF patients had the significantly greatest improvement in HRQoL across all instruments (p ≤ 0.021). Pulmonary fibrosis had similar improvements whereas pulmonary hypertension had the smallest improvements.				
		SF12-PCS (MCID = 5), average change (IQR): (p < 0.001) COPD 15.9 (11.5, 20.3), Pulmonary arterial hypertension 7.9 (1.0, 14.7), CF 23.8 (19.5, 28.1), Pulmonary fibrosis 13.8 (11.9, 15.8)				
		SF12-PCS (MCID = 5), average change (IQR): (p = 0.020) COPD 2.7 (-0.9, 6.4), Pulmonary arterial hypertension 0.1 (-5.6, 5.7), CF 10.3 (6.4, 14.1), Pulmonary fibrosis 4.8 (3.1, 6.6)				
		AQ20-R (MCID = 1.75), average change (IQR): (p = 0.021) COPD 7.7 (6.4, 9.1), Pulmonary arterial hypertension 4.5 (2.1, 6.9), CF 9.4 (8.2, 10.6), Pulmonary fibrosis 7.9 (7.3, 8.6)				
		EQ5D (MCID = 0.06), average change (IQR): (p = 0.003) COPD 0.15 (0.08, 0.21), Pulmonary arterial hypertension 0.07 (-0.05, 0.19), CF 0.30 (0.22, 0.32), Pulmonary fibrosis 0.16 (0.13, 0.19)				
		No significant difference was identified across all HRQoL instruments between patients who had improved HRQoL and patients who did not have improved HRQoL in CF patients, or patients without a diagnosis of CF (pulmonary fibrosis, COPD, pulmonary hypertension).				
		Diagnosis and quality of life (mean ± SD)				
		SF12-PCS (not improved frequency [% of not improved], improved frequency [% of improved]): COPD 7 (23%), 16 (15%), Pulmonary arterial hypertension 2 (7%), 3 (3%), CF 3 (10%), 11 (10%), Pulmonary fibrosis 18 (60%), 80 (73%)				
		SF12-MCS (not improved frequency [% of not improved], improved frequency [% of improved]): COPD 21 (23%), 2 (4%), Pulmonary arterial hypertension 4 (4%), 1 (2%), CF 8 (9%), 6 (13%), Pulmonary fibrosis 60 (65%), 38 (81%)				
		AQ20-R (not improved frequency [% of not improved], improved frequency [% of improved]): COPD 1 (6%), 22 (18%), Pulmonary arterial hypertension 3 (19%), 2 (2%), CF 0 (0%), 14 (11%), Pulmonary fibrosis 12 (75%), 87 (70%)				
		EQ5D (not improved frequency [% of not improved], improved frequency [% of improved]): COPD 14 (23%), 9 (11%), Pulmonary arterial hypertension 5 (8%), 0 (0%), CF 3 (5%), 11 (14%), Pulmonary fibrosis 38 (63%), 61 (75%)				
2020	Perez^25^	Assessments and multi-instrument HRQoL battery	Before and at 3- and 6-months post lung transplantation	Yes	Patients on waiting list	-
		Post lung transplantation, CF patients experienced improvements in HRQoL. At 6-months post lung transplantation: SF12-PCS (MCID = 5) improved by 33.0 (95% CI, 28.9, 37.1), 6.6-fold what is clinically significant, SF12-MCS (MCID = 5) improved by 7.6 (95% CI, 3.7, 11.5), 1.5-fold what is clinically significant, and AQ20-R (MCID = 1.75) improved by 12.41 (95% CI, 11.19, 13.64), 7.1-fold what is clinically significant. The range of improvement overall was from 1.5 to 7.1-fold the instrument's clinically significant difference.				
		HRQoL instrument scores at baseline, 3-months post Lung transplantation, 6-months post Lung transplantation (mean ± SD)				
		SF12-PCS (MCID = 5): 21.0 ± 7.2, 51.1 ± 8.8, 54.5 ± 8.4				
		SF12-MCS (MCID = 5): 46.3 ± 7.5, 56.7 ± 8.3, 53.5 ± 9.4				
		AQ20-R (MCID = 1.75): 12.00 ± 1.98, 3.37 ± 2.43, 2.58 ± 2.01				
		EQ5D (MCID = 0.06): 0.56 ± 0.29, 0.90 ± 0.09, 0.90 ± 0.16				
2020	Stacel^26^	Questionnaire administered at follow-up clinic	Pretransplant, 3-, 6- and 6-monthly thereafter until 36-months. 54.97 ± 41.28 months (range: 12-months to 145-months)	Yes	Patients with other diagnoses (non-CF), Normative polish population	‒
		When compared to a healthy Polish population, patients who have undergone lung transplantation have improved HRQoL in the WHOQoL-BREF domains: physical, psychological, environmental (26.75 points ± 4.97 vs. 15.79 points ± 2.23; 22.28 points ± 3.52 vs. 13.79 points ± 2.51; and 29.77 points ± 3.72 vs. 13.10 points ± 2.43, respectively).				
		In all domains of the WHOQoL-BREF, no statistically significant difference was identified between CF patients when compared to COPD and ILD patients.				
		WHOQoL-BREF scores comparing CF, COPD, and interstitial lung disease patients:				
		Somatic: CF 27.53 ± 4.53, COPD 26.93 ± 4.68, ILD 26.75 ± 4.28				
		Psychological: CF 23.20 ± 2.57, COPD 21.53 ± 3.18, ILD 22.90 ± 3.59				
		Social: CF 11.53 ± 2.75, COPD 11.13 ± 2.44, ILD 11.30 ± 2.97				
		Environment: CF 30.40 ± 2.53, COPD 28.73 ± 3.15, ILD 30.40 ± 4.19				
		CF patients had significantly improved SGRQ scores compared to ILD in the domains: total score (p = 0.028) and activity (p = 0.025). SGRQ scores were markedly improved in CF patients than in ILD and in COPD patients, but no statistically significant difference was identified.				
		SGRQ scores comparing CF, COPD and interstitial lung disease patients:				
		Symptoms (%): CF 23.28 ± 21.16, COPD 36.99 ± 32.47, ILD 32.36 ± 23.77				
		Activity (%): CF 20.28 ± 26.25, COPD 40.26 ± 30.77, ILD 39.26 ± 21.81				
		Impacts (%): CF 17.67 ± 16.84, COPD 28.21±18.13, ILD 28.65 ± 16.66				
		Total (%): CF 19.38 ± 17.51, COPD 33.07 ± 23.39, ILD 32.47 ± 16.02				
		Comparing WHOQoL-BREF scores to a Polish normative population (Jaracz et al.) the CF patients had improved HRQoL in the domains: somatic (27.53 ± 4.53 vs. 15.79 ± 2.23), psychological (15.79±2.23 vs. 23.20 ± 2.57) and environmental (30.40 ± 2.53 vs. 13.10 ± 2.43), but no statistically significant difference was identified.				
		The CF patients post lung transplantation had improved HRQoL using the SGRQ instrument when compared to a normative CF population from a Toronto Centre Study (19.4% vs. 46.0%)				

AQ20-R, Airways Questionnaire 20 – revised; BMI, Body Mass Index; CFQoL, The Cystic Fibrosis Quality of Life; COPD, Chronic Obstructive Pulmonary Disease; CF, Cystic Fibrosis; CFB, Cystic Fibrosis and Bronchiectasis; EQ5D, EuroQol 5D; FEV1, Forced Expiratory Volume in the first second; HRQoL, Health Related Quality of Life; ILD, Interstitial Lung Disease; IPF, Idiopathic Pulmonary Fibrosis; IQR, Interquartile Range; LAS, Lung Allocation Score; MCS, Mental Component Summary Score; NHP, Nottingham Health Profile; OAD, Obstructive Airways Disease; PCS, Physical Component Summary score; QALY, Quality of Life Years; SF12, 12-item Short Form survey; SF-36, 36-item Short Form survey; SD, Standard Deviation, SGRQ, St. George Respiratory Questionnaire; USA, United States of America, WHOQoL-BREF, World Health Organisation Quality of Life Brief Questionnaire.

### Synthesis of results

Qualitative analysis was performed according to previous guidelines where HRQoL outcomes were categorized into physical, emotional, social, and functional health domains which were either disease-specific or generic.

### Risk of bias

The risk of bias in individual studies was assessed by a qualitative analysis based on study quality and data tabulated in Table 1. Given that meta-analysis was not feasible because of clinical heterogeneity between the studies, specific tools were not utilized to assess bias within each study. Each study was assessed for significant selection, performance, detection, or reporting bias. This is supported by the Cochrane guidelines on systematic reviews^23^ and an assessment of bias was also performed according to the PRISMA guidelines.^13^, ^14^, ^15^ Previously outlined guidelines (NHMRC Evidence Hierarchy) were used to assess levels of evidence for individual studies.^24^

## Results

### Study Selection

Following a thorough literature search, ten studies were chosen to be included in this systematic review (Fig. 1). The studies included demonstrated significant diversity between comparison groups, used a wide variety of HRQoL instruments, lacked pre-operative data, and occasionally did not express data as mean ± standard deviation. Given the lack of clinical, statistical, and methodological heterogeneity, meta-analysis and direct comparison of the included studies were precluded.

### Study characteristics and risk of bias within studies

A systematic review of all literature meeting the eligibility criteria (Fig. 1.) through the aforementioned databases was employed to minimize reporting bias. Systematic analysis of the resulting studies was undertaken to determine the strength of evidence and is reported in Table 1. The scarcity of comprehensive results and heterogeneity in data presentation precluded detailed and direct comparisons of the included studies. Variations in study design and statistical analysis additionally limited the comparison of results. The preclusion of direct and comprehensive comparison represents a source of bias as heterogeneity between studies alters the degree to which exact conclusions can be made.

A total of ten studies, 1,494 patients, were included in this systematic review, and cohort size ranged from 23 to 387 patients. Rates of follow-up varied between studies; length of follow-up ranged between three months postoperatively^25^ to 117 months post-LTx,^26^ most studies did not report mean follow-up length with one study not reporting follow-up entirely. Response rates were variable and poorly reported. Five studies reported response rates that ranged between 20.9%^27^ and 87%,^28^ these studies provided no interpretation or reasoning for their response rates. Studies lacking response rates provided no reason as to why this data was not provided. Patients who are unable to respond may tend to be more unwell and as such low response rates may skew data more positively.

Two studies reported results from the “Breath Again” study.^25^^,^^29^ All included studies were selected based on the use of HRQoL instruments validated for patients post-LTx or with chronic pulmonary disease. Instruments were either pulmonary-specific, disease-specific, or generic. Seven studies used generic questionnaires. Four studies used the 36-Item Short Form Survey (SF-36)^28^^,^^30^, ^31^, ^32^ two of which in addition to the pulmonary-specific St. George Respiratory Questionnaire (SGRQ).^28^^,^^31^ Two studies used both the EuroQol 5D (EQ-5D) questionnaire, the 12-Item Short Form Survey (SF-12), and the pulmonary-specific Airways Questionnaire 20 – Revised (AQ20-R).^25^^,^^29^ One study used the Nottingham Health Profile alone,^27^ and one study used the WHOQOL-BREF questionnaire^26^ in conjunction with the SGRQ.^26^ Two studies used the pulmonary disease-specific Cystic Fibrosis Quality of Life (CFQoL) questionnaire,^33^^,^^34^ both studies did not employ another HRQoL instrument.

### Changes in HRQoL in CF patients through the transplant process

Six studies included in this review reported changes in HRQoL scores of CF patients at various points along the transplant process.^25^^,^^27^^,^^30^^,^^31^^,^^33^^,^^34^ All studies reported at least one score post-transplant and compared scores to waitlisted or non-waitlisted patients. All six studies concluded that LTx significantly improved HRQoL in CF patients relative to either pretransplant scores or equivalent waitlisted patients.^25^^,^^27^^,^^30^^,^^31^^,^^33^^,^^34^

Studies using the disease-specific CFQoL instrument revealed immediate significant improvements in either all nine domains of HRQoL^34^ or the domains of physical functioning, social functioning, treatment issues, and chest symptoms.^33^ Lung recipients tested with the NHP see significant improvements in mobility.^27^ No improvement was seen in the SF-36 MCS,^30^ improvement was seen in the SF-12 MCS.^25^

Considering the first-year post-transplantation, HRQoL was either retained or improved as reported by Perez, Vermeulen, and Copeland et al.^25^^,^^27^^,^^30^ With a significant increase from baseline across SF-12 PCS and MCS, AQ20-R, and EQ5D questionnaires, the patients included in the “Breath Again” study demonstrated continued improvements in HRQoL up to six months post-transplant.^25^ Per Copeland et al. patients with CF immediately post-LTx achieve significant improvements in the physical domains of HRQoL as measured by the SF-36 instrument.^30^ Furthermore, Copeland et al. report retained improvements in physical HRQoL over the first-year post-transplantation with HRQoL scores comparable to the US general population.^30^ Per Vermeulen et al. pretransplant CF patients experience HRQoL limitations in the domains of mobility and energy compared to the general population reference values.^27^ Thus, HRQoL improvements in the first-year post-transplant can be realized in the aforementioned two domains.

Beyond the first year and up to 31 months post-transplantation CF patients do not experience HRQoL scores significantly below the population reference value in any domain of the NHP.^27^ L.G. Singer et al. presented the predicted proportion of time spent in a given health state as measured by either the SF-36 PCS or SGRQ over the five-year period post-transplantation.^31^ Health states were defined by LG Singer et al. using a Markov model as “Much better”, “Better”, “Same/Worse”, and “Dead” each denoting a ten/eight, five/four, zero or less point change, or death, respectively, over the last measured pretransplant SF-36 PCS/SGRQ score.^31^ As measured by the SF-36 PCS, CF patients spent 3.00 years in a “Much Better” state, 0.33 years in a “Better” state, 0.45 years in the “Same/Worse” state, and 1.22 years “Dead”.^31^ Similarly, as measured by the SGRQ, CF patients spent 3.53 years in a “Much Better” state, lower, unreported amounts of time in a Better” or “Same/Worse” state, and 1.22 years “Dead”.^31^

Compared to non-waitlisted patients, lung-recipient CF patients showed significantly better HRQoL in the CFQoL questionnaire domain of treatment issues, all other compared domains showed no significant difference.^34^

### Factors impacting the quality of life in CF patients post-LTx

Two studies placed particular emphasis on factors affecting HRQoL in CF patients post-transplantation.^25^^,^^33^ Gee et al. utilized both simple linear regression and forward selection multiple regression analyses to identify the association between clinical variables and changes in the nine domains of HRQoL measured by the CFQoL instrument.^33^ As the study included waitlisted and non-waitlisted patients both analyses established the percent predicted Forced Expiratory volume in one second (FEV_1_%) as a significant explanatory variable for all domains of the CFQoL.^33^ Further variables identified include age which negatively impacted the domains of physical functioning and career concerns; and female sex, which was associated with worse HRQoL with regard to chest symptoms and emotional functioning and greater HRQoL in the domain of body image.^33^ Additionally, fitting of an access device worsened all domains in simple linear regression analysis and was associated with diminished HRQoL in the domains of body image and career concerns under regression model analysis.^33^

Perez et al. identify a significant positive relationship between FEV_1_% and HRQoL across all utilized tools.^25^ Frailty, as assessed by a short physical performance battery, was negatively associated with the mental component of the SF-12 questionnaire, as well as the EQ5D.^25^ The study refutes BMI as a clinically meaningful variable that results in diminished HRQoL.

### HRQoL outcomes post-LTx compared to non-CF patients

Seven of the ten included studies compared HRQoL scores of patients with CF to patients with other diagnoses. At subsequent follow-ups patients diagnosed with CF returned higher HRQoL scores following lung transplantation.^26^, ^27^, ^28^, ^29^, ^30^, ^31^, ^32^ All seven studies concluded that patients that had received lung transplantation for CF had greater outcomes post-operatively in at least one domain compared to patients who had undergone transplantation for other indications.^26^, ^27^, ^28^, ^29^, ^30^, ^31^, ^32^ Between studies there was variation in the extent to which HRQoL scores differed between CF and non-CF patients within individual domains of scoring instruments and between the overall result of HRQoL instruments.^26^, ^27^, ^28^, ^29^, ^30^, ^31^, ^32^

Vasiliadis et al. reported diagnosis of CF or bronchiectasis, when compared to patients with obstructive airway disease, was significantly associated with improved scores in seven of eight HRQoL domains assessed by the SF-36 instrument, greater scores were not found in the domain of physical functioning.^32^

A 2004 study by Vermeulen et al. aimed to identify differences in HRQoL between CF and non-CF patients from pre-transplantation through to 31 months postoperatively.^27^ In investigating variations in the six domains of HRQoL assessed by the NHP, the study established that CF patients experience significant improvements relative to non-CF patients in the domains of mobility at four months, and sleep at four-, seven-, and 13 months post-transplant.^27^ The remaining four domains revealed no significant difference between CF and non-CF patients at any time point post-transplant.^27^

Three studies utilizing the pulmonary disease-specific SGRQ HRQoL instrument established significant differences between mean group scores when evaluated with respect to diagnosis.^26^^,^^28^^,^^31^ Patients with CF returned the highest HRQoL scores and showed significant differences when compared to patients with interstitial lung disease.^31^ Patients diagnosed with CF achieve significantly greater HRQoL as measured by the AQ20-R instrument.^29^

## Discussion

### Summary of evidence and interpretation

The main findings of the studies included in this systematic review are that (i) Lung-transplantation results in improvements in HRQoL in CF patients relative to their baseline waitlisted state, (ii) Up to five years postoperatively CF patients retain their HRQoL at levels similar to the general population, (iii) Compared to equivalent non-waitlisted CF patients transplant recipients achieve similar levels of HRQoL, except in the domain of treatment issues where HRQoL is higher, (iv) There are several modulating factors that influence HRQoL outcomes in CF patients post-LTx, and (v) Compared to lung recipients with other diagnoses CF patients achieve either greater or equal levels of HRQoL.

Accurately quantifying preoperative HRQoL is imperative in evaluating and describing the effect of LTx on health-related quality of life in patients with CF. The cohort of patients with CF undergoing lung transplantation shows significant demographic variation from those with other disease indications.^26^, ^27^, ^28^, ^29^, ^30^, ^31^, ^32^ Patients are younger, have lived with their disease for a lifetime, and often have extrapulmonary manifestations of their disease.^26^, ^27^, ^28^, ^29^, ^30^, ^31^, ^32^ Debska et al. using the disease-specific CFQoL instrument reported all domains to be within one standard deviation of normal, however, the mean scores of several domains were also within the range of poor quality of life.^34^ As evident, evaluating change in HRQoL as a result of LTx reflects considerable complexity. Burker et al. and Parsons state that pretransplant CF patients do not compare themselves to a situation of perfect health.^35^^,^^36^ Thus, any statistically significant improvement in HRQoL from baseline could be considered beneficial.

Compared to waitlisted CF patients, lung recipients saw statistically significant improvements in all domains of the CFQoL as measured by Debska et al.^34^ Similarly, patients evaluated with the generic NHP, or SF-36 questionnaires revealed specific improvements in physical domains of HRQoL within the first-year post-transplant and showed retained global HRQoL up to 31 months post-transplant at levels similar to the general population.^25^^,^^27^^,^^30^^,^^31^^,^^33^^,^^34^ Although the accurate evaluation of HRQoL prior to transplantation remains questionable, the statistically significant improvements in HRQoL reflect the benefit CF patients may have on the average gain from LTx.^25^^,^^27^^,^^30^^,^^31^^,^^33^^,^^34^ Furthermore, evaluation of CF patients post-LTx relative to non-waitlisted counterparts establishes further benefits to transplantation.^33^^,^^34^ However, Gee et al. state that these differences may be due to heterogeneity in the non-waitlisted group, which consisted of stable patients, in addition to individuals who were either not medically appropriate for transplantation or those who refused transplantation.^33^

Indication for LTx in CF patients can be determined by FEV_1_%, with FEV_1_% < 30% reflecting a definite indication in adults for referral to a LTx center.^8^ The latest estimate for survival post-LTx for CF patients is 9.9 years,^37^ however, survival of patients with FEV_1_% < 30% during periods of clinical stability is estimated at around 6.6 years,^38^ this value may continue to increase as patients with advanced-disease benefit from new disease-modifying medications.^4^ Thus, understanding current estimates of HRQoL for patients with CF may play a significant role in distinguishing treatment options given the benefits of LTx.

Although waitlisted patients may report HRQoL within ranges of the general population,^27^^,^^34^ improvements in HRQoL post-LTx relative to baseline states reflect that, if patients have an objectively reduced lung function as measured by FEV_1_%, lung transplantation continues to represent a treatment modality that empirically improves patient well-being. Thus, as there continue to be improvements in the medical management of CF, the current determination of waitlisting CF patients for transplantation continues to represent an objectively quantifiable indication for transplantation.

Demographic, clinical, and disease-related factors associated with CF patients have been shown to impact the domains of HRQoL.^25^^,^^33^ Gee et al. identified elderly patients have reduced physical functioning and greater career concerns.^33^ Gee et al. cite the cultural desirability for a lean body shape in females as opposed to a heavier body shape in males as the reason for the reduced quality of life associated with body image in males.^33^ Furthermore, the use of an access device was associated with significantly poorer career concerns and body image.^33^ An investigation is required into whether the site of insertion of the access device or its presence resulted in poorer career concerns and body image.

Frailty, as measured by a Short Physical Performance Battery (SPPB), was associated with a reduced SF-12 MCS and EQ5D score.^25^ Frailty, as a reversible process, represents a novel target for intervention to improve HRQoL post-LTx.^25^ Thus, clinicians may consider encouraging interventions to improve frailty such as home-based exercise regimens, pulmonary rehabilitation, physical therapy, and improved nutritional intake.

Compared to patients with other end-stage pulmonary diseases, patients with CF experience the greatest improvement in HRQoL post-LTx.^26^, ^27^, ^28^, ^29^, ^30^, ^31^, ^32^ Demographically matched patient cohorts reveal that CF patients overall achieve greater HRQoL from the immediate postoperative period to up to three years after transplantation in several domains across HRQoL instruments.^26^, ^27^, ^28^, ^29^, ^30^, ^31^, ^32^ Given the greater levels of HRQoL experienced by CF patients, they represent an ideal cohort to further establish interventions to improve HRQoL in other disease groups.

The medical management of CF patients has witnessed substantial evolution over the last decade with the advent of CFTR modulators.^8^^,^^10^^,^^11^ As such, the outcomes following surgical management of CF patients via lung transplantation must be reassessed.^10^ The current evidence presents lung transplantation as an immediate and sustained benefit to HRQoL over pretransplant individuals with advanced-stage pulmonary disease.^25^^,^^27^^,^^30^^,^^31^^,^^33^^,^^34^ Furthermore, the evidence reveals the benefit seen over patients undergoing transplantation for other diagnoses^26^, ^27^, ^28^, ^29^, ^30^, ^31^, ^32^ in addition to factors that improve or impair HRQoL.^25^^,^^33^

### Review limitations

Limitations of this systematic review were the lack of published evidence on post-LTx HRQoL in CF patients, the significant clinical, methodological, and statistical heterogeneity of included studies, and the poor robustness of the included studies. A thorough review of all literature on the topic resulted in the inclusion of ten studies of 1,494 patients, reflecting high statistical power. However, individual studies either analyzed small numbers of patients with CF or were themselves limited in cohort size, with Gee et al. analyzing 223 CF patients, as such limiting the ability to draw extensive conclusions from the data.

Three of the ten studies focussed specifically on CF patients, with two using disease-specific HRQoL instruments, as opposed to generic or pulmonary-specific instruments used by the remaining studies. Overall, the findings tended to be broadly consistent across instrument types, with the instruments largely showing improvements in HRQoL. However, the results of the studies using varying instrument types are minimally comparable, increasing the heterogeneity of included studies. For instance, the domains of the SGRQ report symptoms, activity, and impact,^19^^,^^20^ and the SF-36 represents HRQoL through mental and physical domains.^20^^,^^21^ However, all included instruments have been validated for lung transplant patients. Patients were studied at highly variable time points both pre- and post-transplant. The statistical heterogeneity involved comparison to other diseases, pretransplant state, non-waitlisted patients, or factors impacting HRQoL with analysis varying from multiple linear regression to *t*-tests.

The included studies are considered NHMRC level III or IV evidence and as such reflect poor robustness and increased risk of bias. However, utilization of research methodology considered NHMRC level II evidence, such as a randomized controlled trial design would be impractical or unethical in answering the clinical question at hand. Albeit this review represents the current state of available evidence and best summarises the HRQoL outcomes post-LTx in patients with CF.

## Conclusion

The management of CF reflects an evolving field. Disease-modifying CFTR modulators represent a significant advancement in the medical management of the disease and will lead to substantial changes in the pretransplant patient cohort. The importance of this review lies in four key assertions. Firstly, the review provides a clear view of the current impact of lung transplantation on HRQoL outcomes for CF patients with advanced-stage pulmonary disease. Secondly, as further evidence on longer-term HRQoL outcomes in medically managed CF patients continues to emerge this review provides a clear baseline benefit of lung transplantation from which future changes can be measured. Thirdly, this review quantifies the baseline level of improvement allocated by LTx to CF patients, thus, clinicians have a standard to which emerging evidence on longer-term HRQoL outcomes in medically managed CF patients can be compared. Finally, although the use of CFTR modulators is increasing there remains a large cohort of patients who either have or are progressing to advanced-stage disease, whether that be due to a lack of access to treatment or inefficacy of medical management and will require lung transplantation. The findings of this systematic review aim to enable greater clinical judgment with regard to the available medical and surgical treatment options and enable future research to better treat patients with CF.

## Abbreviations

AQ20-R, Airways Questionnaire 20 – Revised; CF, Cystic Fibrosis; CFQoL, The Cystic Fibrosis Quality of Life Questionnaire; CFTR, Cystic Fibrosis Transmembrane conductance Regulator gene; EQ-5D, EuroQol-5D; HRQoL, Health-Related Quality of Life; LTx, Lung Transplant; MCS, Mental Component Score of the SF-36; NHMRC, National Health and Medical Research Council; PCS, Physical Component Score of the SF-36; FEV_1_%, Percent predicted forced expiratory volume in one second; PRISMA, Preferred Reporting Items for Systematic Reviews and Meta-Analyses; SF-36, Medical Outcomes Survey Short-Form-36; SGRQ, St. George Respiratory Questionnaire; SPPB, Short Physical Performance Battery.

## Authors' contributions

The authors confirm their contribution to the paper as follows: study conceptualization and methodology: A.R. and A.S.; data curation: A.R. and D.J.; formal analysis, investigation and validation of results: A.R.; writing - original draft: A.R. Writing - review & editing: all authors.

## Funding

This research received no funding.

## Conflicts of interest

The authors declare no conflicts of interest.

## References

[1] Goldman S, Sethi GK, Holman W, Thai H, McFalls E, Ward HB (2011). Radial artery grafts vs saphenous vein grafts in coronary artery bypass surgery: a randomized trial. Jama.

[2] Katkin JP. (December. 2012). Cystic fibrosis: Clinical manifestations and diagnosis. Tilgjengelig på.

[3] Yeung JC, Machuca TN, Chaparro C, Cypel M, Stephenson AL, Solomon M (2020). Lung transplantation for cystic fibrosis. J Heart Lung Transplant.

[4] Turcios NL. (2020). Cystic fibrosis lung disease: an overview. Respir Care.

[5] Braun AT, Merlo CA. (2011). Cystic fibrosis lung transplantation. Curr Opin Pulm Med.

[6] Chambers DC, Perch M, Zuckermann A, Cherikh WS, Harhay MO, HayesJr D (2021). The International Thoracic Organ Transplant Registry of the International Society for Heart and Lung Transplantation: thirty-eighth adult lung transplantation report ‒ 2021; focus on recipient characteristics. J Heart Lung Transplant.

[7] Yeung JC, Keshavjee S. (2014). Overview of clinical lung transplantation. Cold Spring Harbor Perspecti Med.

[8] Kapnadak SG, Dimango E, Hadjiliadis D, Hempstead SE, Tallarico E, Pilewski JM (2020). Cystic Fibrosis Foundation consensus guidelines for the care of individuals with advanced cystic fibrosis lung disease. J Cyst Fibros.

[9] Koutsokera A, Varughese RA, Sykes J, Orchanian-Cheff A, Shah PS, Chaparro C (2019). Pre-transplant factors associated with mortality after lung transplantation in cystic fibrosis: a systematic review and meta-analysis. J Cyst Fibros.

[10] Benden C, Schwarz C. (2021). CFTR modulator therapy and its impact on lung transplantation in cystic fibrosis. Pulm Ther.

[11] Pettit RS, Fellner C. (2014). CFTR modulators for the treatment of cystic fibrosis. Pharm Ther.

[12] Shteinberg M, Taylor-Cousar JL. (2020). Impact of CFTR modulator use on outcomes in people with severe cystic fibrosis lung disease. Eur Respir Rev.

[13] Moher D, Altman DG, Liberati A, statement Tetzlaff J.PRISMA (2011). Epidemiology.

[14] Moher D, Liberati A, Tetzlaff J, Altman DG. (2010). Preferred reporting items for systematic reviews and meta-analyses: the PRISMA statement. Int J Surg.

[15] Moher D, Liberati A, Tetzlaff J, Altman DG, Group P. (2009). Preferred reporting items for systematic reviews and meta-analyses: the PRISMA statement. PLoS Med.

[16] Kaplan RM, Ries AL. (2007). Quality of life: concept and definition. COPD.

[17] Killewo J, Heggenhougen K, Quah SR. (2010).

[18] Gee L, Abbott J, Conway S, Etherington C, Webb A (2000). Development of a disease specific health related quality of life measure for adults and adolescents with cystic fibrosis. Thorax.

[19] Jones P, Quirk F, Baveystock C. (1991). The St George's respiratory questionnaire. Respir Med.

[20] Singer J, Chen J, Blanc PD, Leard LE, Kukreja J, Chen H. (2013). A thematic analysis of quality of life in lung transplant: the existing evidence and implications for future directions. Am J Transplant.

[21] Ware JE (2000). SF-36 health survey update. Spine.

[22] Balestroni G, Bertolotti G. (2012). EuroQol-5D (EQ-5D): an instrument for measuring quality of life. Monaldi Arch Chest Dis.

[23] Furlan AD, Pennick V, Bombardier C, van Tulder M. (2009). 2009 updated method guidelines for systematic reviews in the Cochrane Back Review Group. Spine.

[24] Health N, Council MR. (2009).

[25] Perez AA, Hays SR, Soong A, Gao Y, Greenland JR, Leard LE (2020). Improvements in frailty contribute to substantial improvements in quality of life after lung transplantation in patients with cystic fibrosis. Pediatr Pulmonol.

[26] Stącel T, Jaworska I, Zawadzki F, Wajda-Pokrontka M, Tatoj Z, Urlik M (2020). Assessment of quality of life among patients after lung transplantation: a single-center study. Transplantation proceedings.

[27] Vermeulen KM, Ouwens J-P, van der Bij W, de Boer WJ, Koëter GH, TenVergert EM. (2003). Long-term quality of life in patients surviving at least 55-months after lung transplantation. Gen Hosp Psychiatry.

[28] Smeritschnig B, Jaksch P, Kocher A, Seebacher G, Aigner C, Mazhar S (2005). Quality of life after lung transplantation: a cross-sectional study. J Heart Lung Transplant.

[29] Singer JP, Katz PP, Soong A, Shrestha P, Huang D, Ho J (2017). Effect of lung transplantation on health-related quality of life in the era of the lung allocation score: a US prospective cohort study. Am J Transplant.

[30] Copeland CAF, Vock DM, Pieper K, Mark DB, Palmer SM. (2013). Impact of lung transplantation on recipient quality of life: a serial, prospective, multicenter analysis through the first posttransplant year. Chest.

[31] Singer LG, Chowdhury NA, Faughnan ME, Granton J, Keshavjee S, Marras TK (2015). Effects of recipient age and diagnosis on health-related quality-of-life benefit of lung transplantation. Am J Respir Crit Care Med.

[32] Vasiliadis H-M, Collet J-P, Poirier C. (2006). Health-related quality-of-life determinants in lung transplantation. J Heart Lung Transplant.

[33] Gee L, Abbott J, Hart A, Conway SP, Etherington C, Webb AK. (2005). Associations between clinical variables and quality of life in adults with cystic fibrosis. J Cyst Fibros.

[34] Dębska G, Cepuch G, Mazurek H. (2014). Quality of life in patients with cystic fibrosis depending on the severity of the disease and method of its treatment. Postępy Hig Med Dosw.

[35] Burker EJ, Carels RA, Thompson LF, Rodgers L, Egan T. (2000). Quality of life in patients awaiting lung transplant: cystic fibrosis versus other end-stage lung diseases. Pediatr Pulmonol.

[36] Parsons E. (1990). Coping and well-being strategies in individuals with COPD. Health Values.

[37] Bos S, Vos R, Van Raemdonck DE, Verleden GM. (2020). Survival in adult lung transplantation: where are we in 2020?. Curr Opin Organ Transplant.

[38] Ramos KJ, Quon BS, Heltshe SL, Mayer-Hamblett N, Lease ED, Aitken ML (2017). Heterogeneity in survival in adult patients with cystic fibrosis with FEV_1_% <30% of predicted in the United States. Chest.

